# Kinematics of Two Special Endurance Trials: A Methodological Contribution to 400-m Performance

**DOI:** 10.5114/jhk/185155

**Published:** 2024-05-17

**Authors:** Krzysztof Mackala, Rafał Omelko, Dariusz Mroczek, Stefan Szczepan, Andrzej Mastalerz

**Affiliations:** 1 Department of Track and Field, Wroclaw University of Health and Sport Sciences, Wroclaw, Poland.; 2 Department of Biological and Motor Sport Bases, Wroclaw University of Health and Sport Sciences, Wroclaw, Poland.; 3 Department of Swimming, Wroclaw University of Health and Sport Sciences, Wroclaw, Poland.; 4 Department of Biomedical Sciences, Faculty of Physical Education, Józef Piłsudski University of Physical Education in Warsaw, Warsaw, Poland.

**Keywords:** competition, intermittent training, step kinematics, fatigue, blood lactate

## Abstract

This study aimed to determine changes in the kinematics of sprint steps based on progressive muscular fatigue during high-intensity 350-m and 500-m trials. Twelve elite healthy male 400-m sprinters with a minimum of six years of regular sprint training experience were recruited. They were divided into two groups for the experiment: a 350-m and a 500-m trial group. Time and kinematics of sprinting step motion for specific segments, i.e., starting to final stages of each trial, were obtained using the Opto Jump-Microgate optical measurement system. The starting phase of each sprint was defined as the section without muscular fatigue (noF), and the final phase was the sprint under muscular fatigue (onF). Each last 25 m of the 50-m evaluated section containing ten complete running steps was selected for detailed statistical analysis. Various patterns of temporal and spatial variables of sprinting efforts were observed between 350-m and 500-m trials. Each trial result was influenced by significant individual changes (p < 0.05). All variables indicated that the two distances differed significantly in terms of running kinematics. This was confirmed by significant differences in the mean step frequency (p < 0.001), which presented a difference of 11.75%, and the mean step speed (p < 0.001). As a result of these changes, a hierarchical intermittent endurance training pattern was defined. The research concluded that special endurance (intermittent sprints) based on 350 m differed significantly in kinematics from sprints over 500 m. Therefore, it should be assumed that the distance of 350 m is more similar in its kinematics to the 400-m competition. This should encourage coaches and athletes to apply a 350-m distance in training developing special endurance, especially in the pre-competitive and competitive periods.

## Introduction

The 400-m sprint is one of the most demanding athletic events, an intermediate event between the sprint of shorter distance and middle distance runs (Grgic et al., 2019). This event maximally stimulates the anaerobic and aerobic energy systems (Arcelli et al., 2008; Zouhal et al., 2010). In addition, it induces significant changes in the kinematics of the race, especially at the end of the performance (Gorostiaga et al., 2010; Hanon and Gajer, 2009). Therefore, analysing 400-m performance and the runner's individual physiological and kinematical responses to this extreme activity may help optimally prepare and control the training process (Iskra et al., 2017). These actions will contribute to optimally increasing 400-m performance in elite and sub-elite sprinters. One of the main factors determining the effectiveness of the 400-m sprint is the athletes’ speed level (Bergamini, 2011; Brüggemann et al., 1999; Čoh et al., 1995). The abovementioned factor is directly related to the length and frequency of steps and the optimal time of foot contact with the ground (Mackala and Fostiak, 2015; Mackala et al., 2019; Maćkała and Mero, 2013). Numerous studies have shown a lack of consensus on some variables, e.g. step length, specific speed, and anthropometric variables (Bezodis, 2012; Coh et al., 2010; Debaere et al., 2013; Manzer et al., 2016; Mates et al., 2021). In contrast, a stronger correlation was shown between strength and flexibility levels (Viru et al., 2001). The decrease in running speed results from a reduction in step frequency and step length (Vazel, 2011). In addition, factors influencing the decline in the running efficiency include technical errors, e.g., an incomplete rebound of the foot from the ground during the running step, a too short running step, and running on the whole feet or through the heel (Mackala, 2007; Mackala et al., 2019). All these variables are more pronounced as fatigue increases. Sprague and Mann (1983), based on measurements made at the 40^th^ and 380^th^ m of the 400-m sprint, showed significant changes in kinematic variables caused by fatigue at the final stages of the distance. These included step frequency, time of the support phase, speed of movement of the centre of gravity of the body, and horizontal speed of the centre of gravity of the foot before its contact with the ground.

As an explanation, fatigue is often defined as a reduction in the ability of the neuromuscular system to generate force (Hanon and Gajer, 2009; Mendez-Villanueva et al., 2008). In the 400-m sprint, while maintaining muscle activation, its tension declines progressively (Zouhal et al., 2010). The duration of muscle contraction increases with progressive fatigue, which is manifested by prolonged ground contact time of the foot (Chapman, 1982; Sprague and Mann, 1983). The duration of the vibrations also increases, mainly due to the slowing down of the relaxation rate (Bigland-Ritchie et al., 1983). Furthermore, muscle acidosis progresses, reducing force production. As fatigue increases, many biochemical and biophysical changes co-occur (Ward-Smith, 1999). These changes significantly disturb the aforementioned sprinting technique, forcing the coaches and athletes to introduce training interventions limiting this process. Therefore, the main task of sports training in sprinting, with particular emphasis on the 400-m sprint, should be multidimensional activities aimed at increasing motor and technical abilities (Iskra et al., 2017). Hanon and Gajer (2009) stated that 400-m sprinters should achieve a very high running speed applied to a very economical movement structure (sprint step technique) and preserve this optimal step pattern technical efficiency despite increasing muscular fatigue. For these reasons, it seems reasonable to employ a training and control mechanism that will allow to identify areas for biomechanical variable improvement to reach high levels of 400-m performance.

Based on this, the result in the 400-m sprint is determined by the ability to maintain high average speed (minimize muscular fatigue) over the entire distance. The pace distribution over this distance depends mainly on the special endurance predispositions (Hanon and Thomas, 2011; Iskra et al., 2017; Vazel, 2011), directly related to anaerobic changes and blood lactate concentrations (Gefen et al., 2002). In terms of training theory, special endurance describes efforts with a submaximal and maximum intensity in 90–98% of the runner’s abilities and running sections from 300 to 600 m with optimal rest periods (Gorostiaga et al., 2010; Iskra et al., 2016). The application of the training load depends mainly on the period of the annual training cycle (Warden and Britain, 1988).

Therefore, this work aimed to evaluate changes in kinematic variables observed during the initial and final stages of the 350-m and 500-m runs by well-trained 400-m athletes. An additional aspect was to clarify which distance, 350 m or 500 m, was more effective in developing special endurance at the 400-m distance to improve performance. Furthermore, the individual running strategy, the dynamics of changes in kinematic variables, and increased muscle fatigue were considered. Based on all these factors, we hypothesized that 350 m would be a more effective training means for increasing the level of special endurance in the 400-m sprint compared to 500 m. Therefore, we also assumed that 350 m would improve performance in the 400-m sprint to a greater extent than 500 m.

## Methods

### Procedures

The experiment was carried out in May, at the beginning of the competitive period. This allowed us to obtain the optimal disposition of athletes in the context of their readiness for future competition in the 400-m sprint. The research was conducted over two days. On the first day, measurements of somatic variables were carried out. The anthropometric variables of body height (m) and body mass (kg) were taken (Table 1) on a scale with accuracy of 0.01 m and 0.01 kg, respectively. On the second day, on a synthetic track, the kinematics of the 350-m and 500-m running trials were recorded using the OptoJump-Microgate optical measurement system (Optojump, Bolzano, Italy). The results were obtained using FinishLynx photo-finish technology (Lynx System Developers, Inc. Haverhill, MA USA) as part of the international athletics meeting. Athletes were divided into the 350-m and the 500-m performance group. Each participant performed their test trial individually; the start at a distance of 350 m took place from blocks and at 500 m from a high start position, with the assistance of a qualified starter. In each trial, runners applied a very individual, specific pacing strategy focused on maintaining the running speed to maximise performance. Each trial was preceded by a 40–50-min individual warm-up consisting of jogging for 12 min, stretching with an emphasis on lower limb muscles for about 11–15 min, neuromuscular coordination drills (skipping, bounding, hopping, and acceleration), and two to three 80–150 m runs at progressive speed (intensity). In addition, after the 350-m and 500-m tests, each athlete was subjected to plasma lactate concentration evaluation.

**Table 1:** Characteristics of somatic variables of 400-m sprinters divided into a 350-m group and a 500-m group.

Variable	350-m Group n = 6		500-m Group n = 6		Student’s *t*-test		Confidence		
	x̅	s	x̅	s	*t*	*p*	−95%	+95%	*d*
Age (year)	25.33	3.83	23.80	3.70	0.67	0.5190	22.17	27.10	0.40
Body mass (kg)	78.35	7.62	79.50	4.15	−0.30	0.7705	74.82	82.91	0.18
Body height (cm)	184.92	7.20	187.50	6.37	**−**0.62	0.5486	181.63	190.54	0.38
BMI	22.88	1.20	22.70	2.23	0.18	0.8647	20.86	23.10	0.10
Length of the lower limb (cm)	95.50	4.83	98.60	2.72	−1.27	0.2359	94.12	99.69	0.79

### Participants

Twelve elite healthy male 400-m sprinters with a minimum of six years of regular sprint training experience were recruited. Sprinters participating in the experiment were national team members and competed internationally. Participants’ mean (± SD) age, body height, body mass were: 4.56 ± 3.76 years, 186.21 ± 6.78 cm, and 78.92 ± 5.88 kg, respectively, and their 400-m personal best was 46.34 ± 0.80 s. Sprinters were among the 12 best Polish 400-m runners in the senior and junior (19 years old) age categories. Eight athletes participated in the Olympic Games and the European and World Indoor and Outdoor Championships. They were further divided into two groups for the experiment: a 350-m trial group and a 500-m trial group. The main division criterion included individual preferences of runners and their annual training plan agreed upon with the national team coach and the club coach. Each participant was medically approved to participate in training and competition. They had no orthopedic or physiological restrictions or injuries that could affect performance. Before participating, athletes and coaches were informed in detail about the experimental procedures and the possible risks and benefits of the study. They provided written consent to participate in the experiment. Participants were instructed to maintain their usual intake of food and fluids during the study period. Additionally, they were advised to avoid strenuous physical activity and refrain from eating 48 h and 3 h before testing, respectively. The study design was approved by the Ethics Committee of the Polish Track and Field Association (approval code: PZLA – 5/2019; approval date: 12 May 2019).

### Measurement of Kinematic Variables at the 350-m and 500-m Trials

To measure the basic kinematic variables of the running step, i.e., stride length, stride frequency, support phase time, flight phase time, and the time of performing a single step, the OptoJump measuring device was used. It is an optical measuring system that measures ground contact time during the take-off and the flight phase, with accuracy of 1/1000 of a second. OptoJump consists of two slats (dimensions: 100 cm × 3 cm × 4 cm), one responsible for receiving and controlling data, another for their electronic transmission. LEDs on the transmitting crossbar communicate continuously with those on the receiving crossbar. The system detects any communication interruptions and calculates their duration. Several elements can be connected to extend the measured distance and its variables. By collecting primary data, dedicated software records variables related to the athlete's performance while maintaining maximum real-time accuracy.

**Figure 1:**
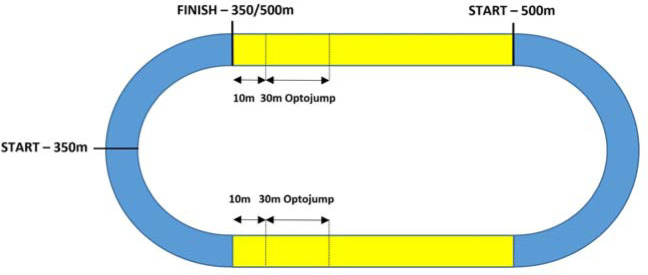
Location of the OptoJump measurement system on the track.

In each trial (350 m and 500 m), kinematic variables of the running step were recorded on two separately measured 30-m sections. For the first 30-m measurement, the OptoJump system was launched at the beginning of the 350-m and the 500-m race. Due to the technical limitations of the OptoJump system, as it cannot be set on a curve, the first (starting) measurement of 30 m for a distance of 350 m took place on a straight line after leaving the curve, i.e., after running 60 m, between 10 and 40 m. To maintain methodological consistency, the first (starting) measurement of 30 m for a distance of 500 m took place on the straight line after running 60 m, i.e., between 60 and 90 m. Therefore, the measurement of the final 30 m for both distances (350 m and 500 m) was marked at the same place; it coincided with the starting phase of the 500-m run, i.e., a straight line between 60 and 90 m. The last 10-m section was excluded from the measurement due to significant disruptions in running technique (measurement values of individual kinematic variables) in the final meters of effort (Figure 1). Ten complete steps performed on each 30-m OptoJump measuring section were subjected to kinematic analysis. To better differentiate between kinematic changes in steps occurring during each 30-m measurement, the following terms were introduced: the initial phase of running without fatigue (noF) and the final phase of running with fatigue (onF).

### Blood Lactate Concentration

The measurements of post-exercise lactate concentration were performed to better understand kinematic changes occurring during 350-m and 500-m test trials. However, only values supporting kinematics of changes with increasing fatigue were considered. Athletes were tested for plasma lactate levels before and after the warm-up, immediately before the test trial, and at the 1^st^, 3^rd^, and 12^th^ min of recovery. Blood lactate concentration (mmol·l^−1^) was determined by the enzymatic method with the Sentinel test (Italia). Arterialised blood was drawn from the fingertip and immediately diluted 10-fold with an excellent isotonic solution containing NaF and NaCl. Lactate concentration was measured in the supernatant obtained after brief centrifugation of the diluted sample. Spectrally pure L-lactate of known concentration and the EPOCH plate reader from BioTek were used as a standard. Measurements were made in duplicate.

### Statistical Analysis

The Shapiro-Wilk test was used to confirm the normality of data distribution in the somatic and kinematic variables (twenty running steps during special endurance trials: 350 m and 500 m). Arithmetic means, standard deviations, coefficients of variation, minimum and maximum values, as well as confidence intervals were calculated. The Student’s *t*-test was used to evaluate the differentiation of the mean values of the somatic and kinematic variables of the running tests. One-dimensional tests for repeated measures, differentiating the kinematic variables of the sprint step between both distances and phases of the run, were used. Additionally, three-way ANOVA with three independent factors, i.e., the measurement segment (MS), the sequence of steps (SS) and the group of competitors (G), was performed. Apparent differences (post hoc tests) between the mean values of variables were calculated using the LSD test (Fisher’s Least Significant Difference). Spearman's rank-order correlation was used to determine the relationship between the selected somatic variables and individual kinematic and lactate concentration values. The significant results are bolded in tables. The level of significance was set at *p* < 0.05.

## Results

Morphological characteristics of 400-m runners did not show significant differences, which indicates that the group of sprinters, despite being divided into two subgroups, was homogeneous (Table 1).

Differences in the length of the covered distance (150 m) between the trials resulted in significant differences in speed, time, and step frequency. However, there were no differences in stride length, which was rather unexpected (Table 2).

**Table 2:** Characteristics of selected kinematic variables of the 350-m and 500-m special endurance running tests.

Variable	350-m Group n = 6		500-m Group n = 6		Student’s *t*-test		Confidence		
	x̄	s	x̄	s	*t*	*p*	−95%	+95%	*d*
Time (s)	40.98	0.73	64.63	1.21	−40.17	**0.0000**	43.41	60.05	23.66
Step length (cm)	223.08	8.34	221.85	14.46	0.18	0.8637	214.87	237.95	0.10
Step frequency (Hz)	4.00	0.12	3.55	0.18	4.99	**0.0007**	3.58	3.99	2.94
Velocity (m/s)	8.90	0.15	8.02	0.67	3.12	**0.0123**	8.09	9.03	1.81

p < 0.05 are in bold

Figure 1 shows the variability of the mean values of several kinematic variables of the sprint changed due to increasing fatigue. During the 350-m run, the measurement of ten steps in the final phase (fatigue) showed an increase in ground contact time by 11.74% compared to the measurement of ten steps in the initial stage (no fatigue). The average flight time increased by 5.57%, while the average step frequency decreased by 7.69%. The decrease in average stride length was 6.78%, and the average speed decreased by 13.82%. In the 500-m run, the increase in the average time of foot contact with the ground was 25.48%. The average flight time increased by 1.68%, while the average step frequency decreased by 11.47%. The average stride length of runners shortened by 11.70%, while the average speed decreased by 21.70%.

**Figure 2:**
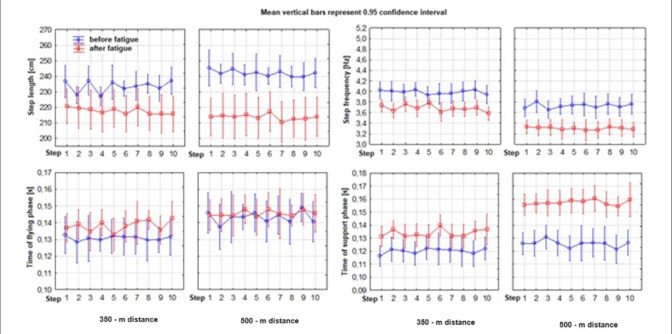
Values of kinematic variables of 10 running steps performed when covering the distance of 30 m with division into a fatigue and a non-fatigue condition of the 350-m and 500-m run.

Figure 2. Values of kinematic variables of 10 running steps performed during the first and the last 30 m of the 350-m and 500-m distance.

The section without fatigue (noF) showed no significant differences between the two runs in almost all five analysed kinematic variables (Table 3). Only the frequency variable showed differences, but not in all steps (1, 3–4, and 7–9). In the ninth step, both the time of the flight phase and the speed of a single step showed significant differences between the 350 m and the 500 m run. The final stage of the run under fatigue (onF) showed significant differences only in three variables: ground contact time, step frequency, and time taken to execute the step. The flight phase time and the indirectly related stride length showed no significant differences between the two runs (350 m and 500 m). This applies to all ten steps performed. The most sensitive kinematic variable of the running step under progressive fatigue was the time of the support phase (at the distance of 350 m, it increased by 11.7%, and at 500 m by 25.5%). It had a crucial impact on the extension of the running cycle and, thus, on the decrease in speed. On the other hand, the flight phase showed the lowest variability during the entire distance and remained relatively constant (the differences were 5.6% and 1.7% at the 350 m and the 500 m run, respectively).

**Table 3:** Comparison of kinematic variables of the ten running steps over the 25-m section performed in the phase with and without fatigue, between the 350-m and the 500-m run; NIR test; probability for post-hoc tests.

Variable	Sequence of steps									
	1	2	3	4	5	6	7	8	9	10
Section no fatigue									
Ground contact time	0.1638	0.4975	0.1263	0.2333	0.9960	0.4789	0.4314	0.3950	0.6247	0.5230
Flying phase time	0.0799	0.2333	0.0935	0.0751	0.0675	0.2217	0.0887	0.1464	**0.0141**	0.2316
Step frequency	**0.0039**	0.0731	**0.0040**	**0.0058**	0.0796	0.0688	**0.0199**	**0.0314**	**0.0048**	0.1050
Step length	0.2265	0.0663	0.2874	0.0624	0.3694	0.2546	0.1979	0.5308	0.3102	0.4777
Step velocity	0.1230	0.8483	0.0623	0.2467	0.3492	0.5541	0.0590	0.2433	**0.0157**	0.3484
	Section – on fatigue.									
Ground contact time	**0.0018**	**0.0074**	**0.0012**	**0.0017**	**0.0007**	**0.0116**	**0.0004**	**0.0018**	**0.0108**	**0.0030**
Flying phase time	0.3067	0.4488	0.2028	0.2825	0.1598	0.1755	0.5359	0.7476	0.1089	0.6902
Step frequency	**0.0008**	**0.0063**	**0.0003**	**0.0009**	**0.0001**	**0.0032**	**0.0007**	**0.0041**	**0.0013**	**0.0082**
Step length	0.3481	0.4892	0.5068	0.8532	0.3992	0.8273	0.1862	0.6431	0.6634	0.7906
Step velocity	**0.0000**	**0.0004**	**0.0000**	**0.0002**	**0.0000**	**0.0029**	**0.0000**	**0.0005**	**0.0001**	**0.0023**

p < 0.05 are in bold

**Table 4:** Three-way ANOVA: parameterization with sigma-restricted, *p* > 0.05.

Effect	MS		G		MS×G		SS		SS×G		MS×SS		MS×SS×G	
	F	*p*	F	*p*	F	*p*	F	*p*	F	*p*	F	*p*	F	*p*
Ground contact time	179.80	**0.0000**	6.11	**0.0355**	14.73	**0.0002**	1.50	0.1620	0.82	0.5991	0.46	0.9700	1.17	0.2931
Flying phase time	37.88	**0.0000**	2.52	0.1467	2.46	0.1135	0.45	0.9005	1.28	0.2579	0.64	0.8679	0.96	0.5050
Step frequency	17.44	**0.0000**	15.63	**0.0033**	4.11	**0.0339**	0.59	0.7983	1.68	0.1067	0.67	0.8338	0.86	0.6263
Step length	38.42	**0.0000**	0.37	0.5593	3.11	0.0691	1.64	0.1183	0.56	0.8246	2.15	**0.0062**	1.72	**0.0401**
Step velocity	163.58	**0.0000**	13.80	**0.0048**	5.62	**0.0127**	1.33	0.2358	2.54	**0.0127**	1.17	0.2948	0.89	0.5959

Measurement section (MS); no fatigue or on fatigue. Group (G); 350-m run or 500-m run The sequence of steps (SS): steps taken in the section; with or without fatigue

**Table 5:** Correlation of Spearman's rank-order (without division into groups) among blood lactate concentrations after the 350-m and the 500-m run, selected kinematic variables and the somatic structure of athletes.

Variable	[1]	[2]	[3]	[4]	[5]	[6]	[7]	[8]	[9]
LA 1 min after the trial [1]	–	−0.25	−0.27	0.07	−0.28	−0.31	**0.62**	0.41	0.07
LA 3 min after the trial [2]	−0.25	–	−0.24	−0.31	−0.21	−0.55	**−0.64**	−0.30	−0.15
LA 12 min after the trial [3]	−0.27	−0.24	–	−0.15	0.42	0.33	−0.31	0.26	−0.10
Step length [4]	0.07	−0.31	−0.15	–	−0.49	0.35	0.02	0.22	**0.82**
Step frequency [5]	−0.28	−0.21	0.42	−0.49	–	0.45	0.17	−0.16	−0.50
Step velocity [6]	−0.31	−0.55	0.33	0.35	0.45	–	0.16	0.05	0.06
Age [7]	**0.62**	**−0.64**	−0.31	−0.10	0.02	0.17	–	0.16	**0.62**
Body mass [8]	0.41	−0.30	0.26	0.22	−0.16	0.05	0.07	–	0.50
Body height [9]	0.07	−0.15	−0.10	**0.82**	−0.50	0.06	−0.14	0.50	–

*p* < 0.05 are in bold

Different characteristics of the studied distances generated different fatigue levels (Table 3), which is supported by changes in particular kinematic variables of the sprint step performed in the final stage of the run. Differences between the 350-m versus the 500-m run were observed in foot contact time (longer by 17.44%), flight time (longer by 5.20%), frequency (lower by 10.33%), stride length (shorter by 1.83%), and speed of a single step (decreased by 11.87%), all to the disadvantage of the 500-m run.

ANOVA of kinematic variables of the running step with three factors, i.e., the measured segment (MS) and the sequence of steps (SS) as independent, and the group of competitors (G) as dependent factors, showed significant differences in the evaluated sections with regard to the running phases with and without fatigue. However, when we analysed the following consecutive steps, there were no significant differences in the variables mentioned above.

According to ANOVA, the interaction between MS and the group of competitors (G): runners at 350 m and 500 m showed significant changes in time of foot contact with the ground, frequency of the step and speed of a single step. On the other hand, when we analysed interactions between the sequence of steps (SS) in both the initial part (no fatigue) and in the final phase (under fatigue) of the run at 350 m and 500 m, no significant differences were observed. In four of the five kinematic variables, the only significant difference was the speed of single step performance. Similarly, when interactions between MS and steps performed in these sections (SS) were analysed, only stride length showed significant changes. Comparing basic kinematic variables among MS, SS, and G, significant differences occurred in only one variable, i.e., step length.

Analysis of Spearman's rank correlation without division into groups (all participants) including lactate concentration levels at the 1^st^, the 3^rd^, and the 12^th^ min after the 350-m and the 500-m run, selected kinematic variables and the somatic structure of athletes, was performed. No relationship was found between lactate levels and kinematic variables. The only significant association was noted between the athlete's age and lactate concentration after the 1^st^ and the 3^rd^ min. This indicates that training experience may correlate positively with post exercise lactate concentration.

## Discussion

This study aimed to determine changes in the kinematics of sprint steps based on increasing muscular fatigue during 350-m and 500-m trials, used as special endurance modalities to enhance 400-m performance. The main conclusion was that special endurance (intermittent runs) over a distance of 350 m differs significantly in kinematics from runs over 500 m. Therefore, the 350-m distance is more similar in its kinematics to the 400-m sprint. Nevertheless, blood lactate concentrations indicated that both distances stimulated the lactate system almost equally.

The difference in the length of both distances increased the duration of exercise by an average of 23.65 s. This indicates that both distances differed significantly in terms of running kinematics, which was confirmed by significant differences in the average step frequency (11.75%, *p* < 0.001) and the average step speed. Despite the shorter running time of 350 m, the average stride length did not show significant differences (0.44%). Possible explanations are the similar body height and equivalent length of the lower limbs of tested athletes. These somatic variables, especially in sprints, significantly affect step length (Salo et al., 2011). Another explanation may be the similar level of strength in the lower limbs of athletes (Mackala and Fostiak, 2015; Nummela et al., 1992), which is related to step length. Additionally, in the 350-m run, difference in step length between the initial (noF) and the final phase (onF) of the run was 6.8%, whereas in the 500-m run, it was 11.7%.

Significant differences were observed in step frequency, which was, on average, 3.99 Hz in the 350-m run and 3.73 Hz in the 500-m run. Considering the same stage of the run (between 50 m and 100 m), the world record holder at 400 m, while breaking the record, achieved a frequency of 3.94 Hz (Miyashiro et al., 2019; Pollitt et al., 2018), which shows that the distance of 350 m is closer to the 400-m sprint than the distance of 500 m. The speed of a single running step also changed, and decreased by approximately 1.30 m/s in the final phase of the run and varied, depending on the step, from 7.72 m/s to 8.20 m/s. It can be stated that these changes were influenced by increasing fatigue (Yousif et al., 2019). Similar relationships were found in the analysis of 400-m sprints (Graubner and Nixdorf, 2009; Grgic et al., 2019). The flight phase time did not change, with a slight tendency to increase at the end of the trial. This may be surprising because, according to the Gundlach's assumption (Ballreich, 1976), the flight phase should shorten as the step length decreases.

The previously mentioned progressive fatigue with increasing distance (350 m vs. 500 m) resulted in significant changes in the support phase of the step. This was evident in the final phase of both runs, where foot contact time with the ground increased by 11.7% and 25.5% in the 350-m and the 500-m run, respectively. Such changes may result from increased activity of the lower limb muscles just before the intended contact with the ground and at the beginning of the transition to the take-off. This was confirmed by Salo et al. (2011) who claimed that in the initial period of foot contact with the ground, energy decreased due to braking and changes in speed. As a result, part of the energy stored in the tissues of the lower limb is released, contributing to raising the center of gravity of the body and, thus, changes in movement (Grimshaw et al., 2010). This requires increased work of actively contracting muscles, mainly due to increasing metabolite levels (Yousif et al., 2019; Wan et al., 2017). Therefore, the duration of the entire support phase is extended.

Changes in kinematic variables caused by increasing fatigue were more visible during the 500-m run. This may be explained by the classification of bioenergetic efforts, as a run over a distance of 350 m (duration of 40.98 ± 0.73 s) can be classified as an anaerobic exercise with glycolytic capacity, while a 500-m run (duration of 64.63 ± 1.21 s) can be classified as an aerobic-anaerobic exercise, where the contribution of anaerobic metabolism is approximately 60% (Plevnik et al., 2013; Vilmi et al., 2016) and the remaining contribution is aerobic metabolism. However, from a practical point of view, comparison of changes in blood lactate concentration is also necessary, although it was not the subject of detailed analysis in this study. Immediately after both trials, a non-significant difference of 2.06 mmol/l (10.3%) in blood lactate concentration between the sprinters' groups was observed. The highest lactate concentration was recorded in the 350-m trial 12 min after the cessation of the run (21.97 mmol/l). Therefore, the distance of 350 m stimulated glycolysis to a greater extent, allowing for higher values of maximum acidification, while the distance of 500 m, despite the initially lower maximum value, required more time for recovery and the body's return to homeostasis (Woodward et al., 2018).

The above statements were confirmed by the analysis of variance, especially the interactions between particular factors. The interaction between step sequence (SS) 1 to 10, regardless of the running phase and distance, showed no difference between kinematic variables. This means that the value of particular variables depends on the running phase and distance. In the final stage of the 500-m run, individual kinematic variables set to the next steps showed no significant changes. The only significant difference was the speed of performing a single step. In the remaining interactions between the section and the steps performed in these sections and among the three groups of factors (MS × SS × G), significant changes occurred in only one variable, i.e., step length. This means that to maintain a similar step structure during special endurance compared to the competitive distance (400 m), the selection of the leg is a crucial element.

One of the limitations of the current study may be the small number of participants (n = 10), although they were the best Polish 400-m athletes. This study was a part of a grater and comprehensive research project within which in addition to kinematics and lactate evaluation, strength measurements under static and dynamic conditions, speed, muscle stiffness, and EMG measurements were performed. In the present paper, we focused only on kinematic changes in the running step under progressive fatigue. From a practical point of view, in future studies, it would be interesting to analyze kinematic changes in the running step in repetitive training (e.g., 6 x 350 m), with a specific intensity (e.g., 80%), and a designated rest interval to evaluate changes in running kinematics considering progressive fatigue.

## Practical Implications

As the distance of special endurance modalities increases, there is a significant and usually unfavorable change (reduction) in step length. This is a negative phenomenon because an incorrect movement pattern acquired during training will be then transferred to competition. Therefore, choosing the optimal running section for developing special endurance in a 400-m race is of paramount importance for coaches and athletes. Based on our results, it can be stated that the distance of 350 m is more similar in its kinematics to the 400-m sprint, and coaches and athletes are recommended to apply this distance in training focused on special endurance, especially in the pre-competitive and competitive periods.
